# Federal Housing Assistance and Chronic Disease Among US Adults, 2005–2018

**DOI:** 10.5888/pcd20.230144

**Published:** 2023-11-30

**Authors:** Christine Kim, Lauren M. Rossen, Bryan Stierman, Veronica Garrison, Craig M. Hales, Cynthia L. Ogden

**Affiliations:** 1 Epidemic Intelligence Service, Centers for Disease Control and Prevention, Atlanta, Georgia; 2 Division of Health and Nutrition Examination Surveys, National Center for Health Statistics, Centers for Disease Control and Prevention, Hyattsville, Maryland; 3 Division of Research and Methodology, National Center for Health Statistics, Centers for Disease Control and Prevention, Hyattsville, Maryland; 4 Office of Research, Evaluation, and Monitoring, Office of Policy Development and Research, US Department of Housing and Urban Development, Washington, DC

## Abstract

**Introduction:**

Housing insecurity is associated with poor health outcomes. Characterization of chronic disease outcomes among adults with and without housing assistance would enable housing programs to better understand their population’s health care needs.

**Methods:**

We used National Health and Nutrition Examination Survey (NHANES) data from 2005 through 2018 linked to US Department of Housing and Urban Development (HUD) administrative records to estimate the prevalence of obesity, diabetes, and hypertension and to assess the independent associations between housing assistance and chronic conditions among adults receiving HUD assistance and HUD-assistance–eligible adults not receiving HUD assistance at the time of their NHANES examination. We estimated propensity scores to adjust for potential confounders among linkage-eligible adults who had an income-to-poverty ratio less than 2 and were not receiving HUD assistance. Sensitivity analysis used 2013–2018 NHANES cycles to account for disability status.

**Results:**

Adults not receiving HUD assistance had a significantly lower adjusted prevalence of obesity (42.1%; 95% CI, 40.4%–43.8%) compared with adults receiving HUD assistance (47.5%; 95% CI, 44.8%–50.3%), but we found no differences for diabetes and hypertension. We found significant associations between housing assistance and obesity (adjusted odds ratio = 1.29; 95% CI, 1.12–1.47), but these were not significant in the sensitivity analysis with and without controlling for disability status. We found no significant associations between housing assistance and diabetes or hypertension.

**Conclusion:**

Based on data from a cross-sectional survey, we observed a higher prevalence of obesity among adults with HUD assistance compared with HUD-assistance–eligible adults without HUD assistance. Results from this study can help inform research on understanding the prevalence of chronic disease among adults with HUD assistance.

**Summary**

**What is already known on this topic?**

Housing insecurity is associated with poor health outcomes, and receiving federal housing assistance is associated with mixed health outcomes in various studies.

**What is added by this report?**

This study used nationally representative data on adults aged 20 years or older during 2005–2018 and showed that almost half of adults assisted by the US Department of Housing and Urban Development had obesity.

**What are the implications for public health practice?**

Results from this study can help inform future research on the relationship between housing assistance and chronic disease.

## Introduction

Housing insecurity is associated with poor health outcomes (1). Federal housing assistance programs aim to prevent housing insecurity by ensuring that more than 10 million program participants do not pay more than 30% of their household income on rent and utilities (2). According to the US Department of Housing and Urban Development (HUD), only about 20% of households eligible for rental subsidies receive federal assistance (2). The average wait time to receive housing assistance ranges from 2 to 8 years, exposing many people to extended periods of homelessness, overcrowded housing conditions, poor-quality neighborhoods, poor access to food and health care, and other hardships (3). Housing instability is associated with poor access to health care and adverse health outcomes because the burden of housing costs limits resources to pay for other needs such as food and preventive health care (4,5).

Receiving federal housing assistance has been associated with mixed health outcomes in various studies. Some research has documented lower blood lead levels among children receiving housing assistance than comparable children not receiving housing assistance and lower odds of psychological distress among adults receiving housing assistance compared with adults who would receive housing assistance in the future (6,7). In contrast, other studies have found a higher prevalence of cardiovascular disease among adults receiving housing assistance in New York City compared with housing-unassisted residents and a higher prevalence of obesity and hypertension among adults receiving housing assistance in Boston compared to other city residents not receiving housing assistance (8,9). Reasons for poor health among people receiving housing assistance may include various factors, such as lower-quality neighborhoods (10) or differences in characteristics among people receiving housing assistance that result from program entrance preferences and selection (11). For example, HUD administers 3 main housing assistance programs: housing choice vouchers, public housing, and multifamily housing. Housing choice vouchers allow recipients to select privately owned housing that meets program requirements, public housing comprises dwellings owned and managed by public housing authorities, and multifamily housing consists of private properties whose owners receive HUD subsidies to provide a percentage of their housing units to HUD recipients at rates below market value (12). Differences in the health status and health care access of HUD-assistance recipients by housing program have been observed (13); these differences may be related to broad neighborhood-level risk factors such as area-level poverty, neighborhood safety, and food and physical activity environments that differ by type of housing program and that influence some chronic disease outcomes (13–15). Additionally, more than 60% of housing authorities have established preferences (eg, elderly, people with disabilities, people experiencing homelessness or domestic violence) for determining entrance into their programs (11). Many of these factors are associated with higher rates of adverse health outcomes (13,16,17).

Descriptive statistics published by HUD underscore that adults receiving HUD assistance have a high prevalence of chronic disease (18). However, analyses that use nationally representative data to assess associations between receiving housing assistance and the prevalence of chronic health conditions have been limited to specific populations (19,20) or self-reported outcomes of overall health status (7), physical activity (21), unmet medical need (5), or comparison groups not representing HUD-assistance–eligible people (22). Understanding the relationship between housing assistance and obesity, diabetes, and hypertension is important because these conditions disproportionately affect people who are at greater risk for housing insecurity, including people with low income (especially women) (23), people from racial and ethnic minority groups, and people with disabilities (13,16,17). The prevalence of specific conditions, such as obesity, diabetes, and hypertension, among adults receiving HUD assistance compared with adults eligible but not receiving HUD assistance, however, has not been estimated.

Characterization of chronic disease outcomes among HUD-assisted adults compared with HUD-assistance–eligible adults without HUD assistance using nationally representative data would enable housing programs to better understand the health care needs of this population uniquely vulnerable to poor health outcomes. We linked National Health and Nutrition Examination Surveys (NHANES) and HUD administrative data to estimate the prevalence of obesity, diabetes, and hypertension and assess the association of these conditions with housing assistance among adults residing in HUD-assisted housing and HUD-assistance–eligible adults, based on income, without HUD assistance at the time of their NHANES examination during 2005–2018.

## Methods

Self-reported survey data and measured examination data were from NHANES, a cross-sectional national survey designed to monitor the health and nutrition of the civilian noninstitutionalized US population (24). NHANES includes a household interview and in-person examinations, with biospecimen collection, conducted in mobile examination centers (24). These surveys cover 2 calendar years in a single survey cycle. Various subgroups have been oversampled over the years, including non-Hispanic Black people, non-Hispanic White people with lower income, Mexican American people before the 2007–2008 cycle, all Hispanic people after 2007–2008, and non-Hispanic Asian Americans since 2011–2012 (25). NHANES data collection was approved by the National Center for Health Statistics (NCHS) Ethics Review Board (26). These analyses included 7 two-year NHANES data cycles (2005–2006 through 2017–2018). From 2005 through 2018, 39,749 adults aged 20 years or older were interviewed (cumulative response rate, 65.7%), and 38,185 participated in the examination component (cumulative response rate, 63.1%) (27).

Data from linkage-eligible 2005–2018 NHANES respondents were linked by the NCHS Data Linkage Program with data from 2000–2019 administrative records of HUD’s largest housing assistance programs — the housing choice voucher program, the multifamily program, and the public housing program — in all 50 states and the District of Columbia. The linked data allow further examination of nationally representative data on the health and well-being of people receiving housing assistance compared with people eligible for housing assistance based on income. Linkage eligibility was based on the NHANES respondent providing consent and sufficient identifying information; eligibility has improved due to changes in approaches for obtaining consent (12,28). Linkage-eligibility rates for sample respondents (aged ≥18 years) who completed the examination component ranged from 78.3% in 2007–2008 to 94.6% in 2017–2018 for the survey cycles included in our analysis, with an average overall rate of 90.6%. The match rate of receiving HUD assistance among linkage-eligible US adults ranged from 10.4% to 13.4% during 2005–2018, with an average of 12.0% (29). Linked people were identified through deterministic and probabilistic linkage methods based on social security number, first name, last name, middle initial, sex, 5-digit zip code of residence, state of residence, and month, day, and year of birth (12). Approval for this linkage was provided by the NCHS Ethics Review Board. Restricted use data are available through the NCHS Research Data Center (30).

### Measures

#### Chronic conditions

Obesity, diabetes, and hypertension among NHANES respondents were ascertained by using physical measures taken during the examination, along with self-reported information for diabetes and hypertension. Obesity was defined as body mass index (BMI, kg/m^2^) of 30.0 or more. Diabetes was defined as self-reported diabetes (on the basis of the question “Have you ever been told by a doctor or health professional that you have diabetes or sugar diabetes?”) or a hemoglobin A_1c_ of 6.5% or more obtained from the examination. Fasting plasma glucose was not included for diabetes identification because of small sample sizes. Hypertension was defined on the basis of cut points recommended by the American Heart Association (31): mean systolic blood pressure ≥130 mm Hg (up to 3 measurements taken during the examination) or mean diastolic blood pressure ≥80 mm Hg (up to 3 measurements taken during the examination), or self-reported current hypertension medication use (on the basis of the question “Are you now taking prescribed medicine for high blood pressure?”).

#### Housing and income metrics used to develop comparison groups

We used receipt and timing of housing assistance to develop comparison groups. HUD-assisted adults were defined as people who linked to 2000–2019 HUD administrative records and received housing assistance at the time of their NHANES examination or at any point 5 years before their examination. We used a period of 5 years before their examination because the average adult stays in HUD housing for 6 years (median stay, 3–5 years), depending on the housing program (32). The comparison group was defined as all adults who were linkage-eligible but were not HUD-assisted adults or eligible for housing assistance based on income at the time of the survey to receive federal housing assistance, with an income-to-poverty ratio (IPR) <2. This comparison group, hereinafter called HUD-unassisted with an IPR <2, comprises HUD-unassisted, low-income households that may face housing insecurity challenges. The NHANES IPR variable is calculated according to US Health and Human Services poverty guidelines and is used as a proxy measure of HUD income limits (33,34). The IPR is calculated by dividing total family income by the poverty threshold. For example, if a family’s total income is $36,500 and the poverty threshold (which varies by the size of the family and age of the members) for that family is $35,801, the IPR is 1.02. A family whose IPR is less than 1 is considered to be living in poverty.

### Covariates

Demographic characteristics collected in NHANES and used in the analysis included the respondents’ sex, age group at the time of their NHANES interview (20–24 y, 25–44 y, 45–64 y, ≥65 y), race and Hispanic origin (Mexican American [oversampled before 2007], non-Hispanic Black, non-Hispanic White, and “Other” [other Hispanic, other races, and non-Hispanic people reporting multiple races]) (25), marital status (married/living with partner, divorced/separated/widowed, never married), education (less than high school diploma, high school graduate/GED [General Educational Development], some college/college graduate), urban–rural classification (residence in a metropolitan statistical area or nonrural area, nonmetropolitan statistical area) (35), health insurance coverage (private, public, none), household participation in the federal Supplemental Nutrition Assistance Program (SNAP) in the preceding 12 months, IPR (IPR <2, IPR ≥2), household size (1 or 2 people, 3–5 people, ≥6 people), NHANES cycle, and calendar period of examination to account for seasonality (November 1–April 30 or May 1–October 31).

### Statistical analyses

**Population.** The study population included linkage-eligible adult respondents (aged ≥20 years) who participated in the 2005–2018 NHANES examination. Analysis for each chronic condition was limited to respondents who had complete data for that condition and had information on IPR. We restricted the analysis to adults aged 20 years or older to better capture completion of education at the time of their NHANES interview and align with the NHANES sampling approach by age group. We excluded pregnant people from all analyses (n = 581). All analyses accounted for the survey’s multistage, complex sampling design and used examination sample weights adjusted for linkage eligibility (nonresponse), using standard weighting domains to reproduce population counts within sex, age, and race and Hispanic origin subgroups (12).

We examined respondent characteristics by housing-assistance status, comparing HUD-assisted adults with HUD-unassisted adults with an IPR <2. To assess whether receiving housing assistance was associated with the prevalence of chronic conditions among adults and to control for differences between HUD-assisted adults and HUD-unassisted adults with an IPR <2, we used propensity-score (22) weighting methods (36).

**Propensity scores.** We estimated propensity scores via a logistic regression model where the binary outcome was HUD assistance status. Factors associated with both housing assistance and chronic conditions were selected as predictor variables, including age, sex, race and Hispanic origin, health insurance coverage, marital status, education, household size, household participation in SNAP, household IPR, examination period, survey cycle, urban–rural classification, and the linkage-eligible sample weight (along with a weight-squared term to account for nonlinearity).

Propensity scores were applied through the strategy of weighting by the odds, with HUD-assisted adults receiving a weight of 1 and the comparison group (HUD-unassisted adults with an IPR <2) receiving a weight of Propensity Score/(1 − Propensity Score) (37). These weights were then multiplied by the linkage-eligibility–adjusted examination sample weight to create a new composite weight, which was used along with strata and primary sampling units in the final models to account for the survey design and sample selection (6,38,39).

We examined propensity-score distributions in the HUD-assisted and HUD-unassisted groups with box plots to assess the overlap between the groups in the probability distribution for HUD assistance (Supplemental Figure 1 [Appendix]).

**Covariate balance.** We assessed covariate balance through standardized bias plots before and after propensity-score weighting (Supplemental Figure 2 [Appendix]). We calculated standardized bias estimates as the difference in population-weighted proportions (or means for continuous variables) between the housing-assistance and comparison groups divided by the standard deviation in the housing-assistance group (39). After propensity-score weighting, all standardized bias estimates were between −0.25 and 0.25, indicating adequate covariate balance (36).

We described linkage-eligible–weighted and propensity-score–weighted characteristics of HUD-assisted and HUD-unassisted groups. We estimated the linkage-eligible–weighted and propensity-score–weighted prevalence of obesity, diabetes, and hypertension among HUD-assisted adults and HUD-unassisted adults with an IPR <2. We then used propensity-score–weighted logistic regression models to estimate the associations between HUD assistance and the prevalence of chronic conditions. All covariates included in the propensity-score model were also included in the outcome models (ie, doubly robust estimation). We considered whether interactions existed by race and Hispanic origin or sex, but chunk tests for the interaction terms were not significant in any model and were not included in the final models. We used complete case analysis for all models, which resulted in less than 5% of missing data overall.

**Sensitivity analyses.** We conducted sensitivity analyses by using 2 additional comparison groups: future HUD-assisted adults and overall HUD-unassisted adults from the propensity-score model (without IPR restriction). We also conducted sensitivity analyses on data restricted to 2013–2018 NHANES cycles (Supplemental Table 1 and Supplemental Figures 3 and 4 [Appendix]). The future HUD-assisted group was defined as respondents who were linked to 2006–2019 HUD administrative records within 5 years after their examination but not during or 5 years before their examination. Although this group may be considered a better comparison because it accounts for various unmeasured factors related to selection for HUD assistance, the sample size was too small to produce reliable estimates and comparisons. We used the 2005–2018 NHANES cycles to examine the relationship between HUD assistance and chronic conditions using the future HUD-assisted group. The future HUD-assisted group was used as the comparison in a sensitivity analysis because of the smaller sample size (n = 360), and the overall HUD-unassisted group was used to include all adults regardless of income eligibility for HUD assistance. We conducted separate analyses of 2013–2018 NHANES cycles to account for disability status because approximately 20% of households receiving housing assistance from HUD include a person with a disability (20). Disability status was defined as a positive response for 1 or more of 6 self-reported questions on serious difficulty hearing, seeing, concentrating, walking, dressing, and running errands (Supplemental Figure 5 and Supplemental Tables 2 and 3 [Appendix]). NHANES cycles before 2013 did not include the complete disability questionnaire. Finally, we conducted sensitivity analysis on the original model (IPR <2) with adjustment for nativity status (born in the 50 US states or Washington, DC, vs born in another country).

**Figure 1:**
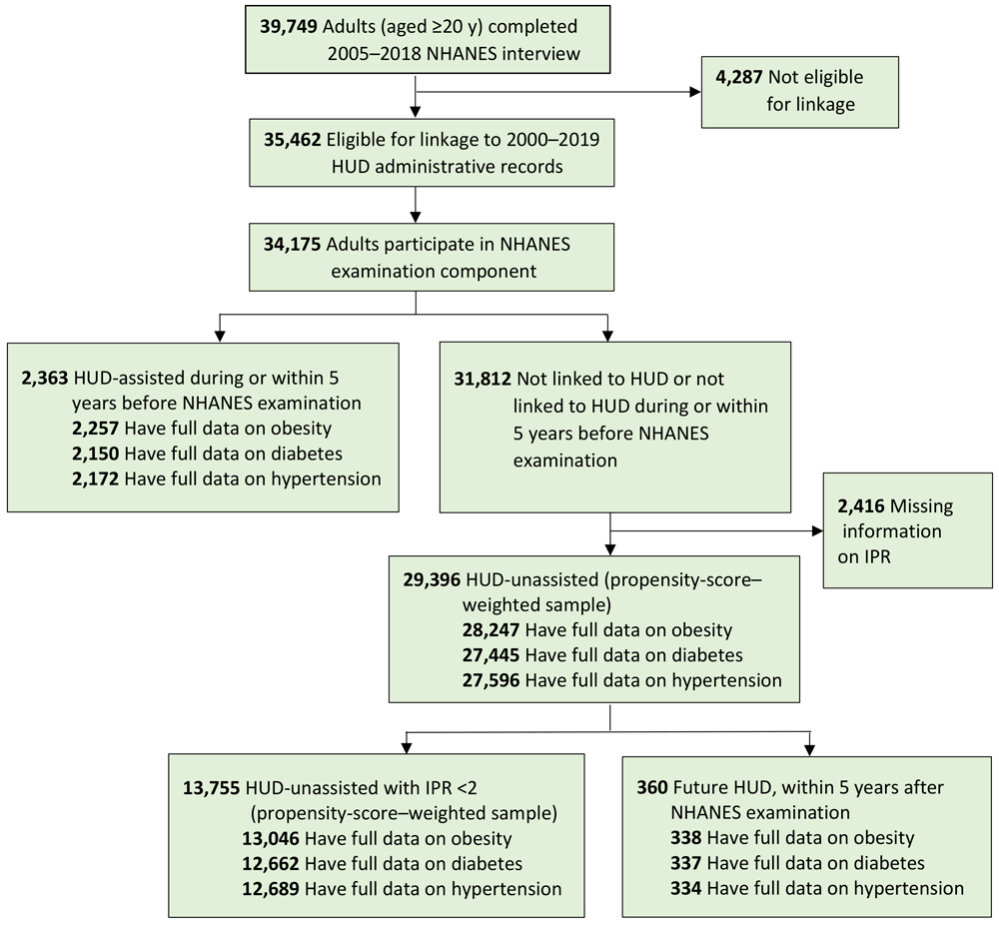
Flowchart of analytic sample, 2005–2018. Data source: National Center for Health Statistics, NHANES, 2005–2018, and linked and linked data from HUD, 2000–2019. Abbreviations: HUD, US Department of Housing and Urban Development; IPR, income-to-poverty ratio; NHANES, National Health and Nutrition Examination Survey.

During the study period (2005–2018), there were 2,363 HUD-assisted adults and 13,755 HUD-unassisted adults with an IPR <2 (Figure 1).

We used χ^2^ tests to test differences between groups at the *P* < .05 significance level. We made no adjustments for multiple comparisons. All statistical analyses were conducted with SAS version 9.4 (SAS Institute Inc) and SAS-callable SUDAAN version 11.0 (RTI International).

## Results

HUD-assisted adults were more likely than HUD-unassisted adults with an IPR <2 to be women (71.2% vs 53.1%), non-Hispanic Black (42.9% vs 13.5%), unmarried (34.2% vs 24.5%) or never married (35.6% vs 22.4%), have less than a high school education (34.6% vs 30.3%), have public health insurance (62.5% vs 40.6%), participate in SNAP (59.7% vs 31.4%), with a household size of 1 or 2 people (45.2% vs 39.0%) (Table **)**. Among HUD-assisted adults, 54.3% received housing choice vouchers, 19.8% lived in public housing, and 25.9% were in multifamily housing programs. After propensity-score weighting, HUD-assisted adults were similar to HUD-unassisted adults with an IPR <2 across all variables except SNAP participation.

**Table:** Linkage-Eligible–Weighted and Propensity-Score–Weighted Demographic and Household Characteristics of US Adults (Aged ≥20 Years), By Housing Assistance Status at the Time of National Health And Nutrition Examination Survey Examination, 2005–2018^a^

Characteristic	Received HUD assistance during or within 5 years before examination, % (95% CI) (n = 2,355)^b^	Did not receive HUD assistance during or within 5 years before examination and had an IPR <2, % (95% CI) [*P* value]			
		Linkage-eligible–weighted (n = 13,182)^b^	*P* value^c^	Propensity-score–weighted (n = 13,182)^b^	*P* value^c^
**Sex**					
Male	28.8 (26.5–31.1)	46.9 (46.0–47.7)	<.001	29.2 (28.0–30.4)	.74
Female	71.2 (68.9–73.5)	53.1 (52.3–54.0)		70.8 (69.6–72.0)	
**Age group, y **					
20–24	20.2 (17.9–22.7)	14.6 (12.9–16.3)	<.001	16.8 (14.8–18.9)	.15
25–44	35.6 (32.5–38.7)	38.4 (37.0–39.9)		36.7 (34.8–38.6)	
45–64	26.3 (24.0–28.6)	28.5 (27.2–29.8)		27.5 (25.9–29.1)	
≥65	17.9 (14.7–21.5)	18.6 (17.3–19.9)		19.1 (17.6–20.6)	
**Race and Hispanic origin^d^**					
Mexican American	6.4 (4.7–8.4)	15.7 (13.3–18.4)	<.001	6.2 (5.0–7.6)	.79
Non-Hispanic Black	42.9 (36.9–49.1)	13.5 (11.7–15.5)		40.8 (36.4–45.3)	
Non-Hispanic White	34.1 (27.4–41.3)	54.9 (51.1–58.7)		34.9 (30.7–39.3)	
Other^e^	16.6 (13.0–20.9)	15.8 (14.1–17.7)		18.1 (15.9–20.5)	
**Marital status**					
Married or living with partner	30.2 (26.0–34.7)	53.1 (51.4–54.8)	<.001	28.6 (26.7–30.4)	.70
Divorced, separated, or widowed	34.2 (30.7–37.9)	24.5 (23.2–25.9)		35.6 (33.6–37.8)	
Never married	35.6 (32.2–39.2)	22.4 (20.4–24.4)		35.8 (32.8–38.9)	
**Education**					
Less than high school diploma	34.6 (32.0–37.3)	30.3 (28.5–32.2)	<.001	37.0 (35.1–38.9)	.36
High school graduate/GED	29.3 (26.9–31.8)	28.3 (27.0–29.6)		28.2 (26.6–29.9)	
Some college/college graduate	36.1 (33.5–38.8)	41.4 (39.1–43.7)		34.8 (32.7–36.9)	
**Geography**					
Metropolitan	77.9 (71.4–83.6)	77.6 (72.0–82.5)	.88	80.6 (75.7–84.9)	.28
Nonmetropolitan	22.1 (16.4–28.6)	22.4 (17.5–28.0)		19.4 (15.1–24.3)	
**Health insurance coverage**					
Private	15.6 (13.7–17.7)	26.1 (24.6–27.7)	<.001	12.6 (11.3–14.1)	.05
Public	62.5 (59.3–65.6)	40.6 (38.7–42.5)		65.7 (63.7–67.8)	
None	21.9 (19.5–24.5)	33.3 (31.6–35.1)		21.7 (20.1–23.3)	
**Household participation in SNAP**					
No	40.3 (36.8–44.0)	68.6 (66.7–70.5)	<.001	36.3 (34.2–38.4)	.04
Yes	59.7 (56.0–63.2)	31.4 (29.5–33.3)		63.7 (61.6–65.8)	
**Income to poverty ratio**					
<2	89.2 (87.4–90.8)	—	—	—	—
≥2	10.8 (9.2–12.6)	—	—	—	
**Household size**					
1 or 2 people	45.2 (41.1–49.4)	39.0 (37.3–40.8)	<.001	50.2 (47.7–52.6)	.10
3–5 people	44.4 (40.4–48.4)	46.4 (45.0–47.8)		39.9 (37.8–42.0)	
>6 people	10.4 (8.6–12.4)	14.6 (13.4–15.9)		10.0 (8.8–11.3)	
**HUD-assisted program**					
Housing choice vouchers	54.3 (49.6–59.1)	—	—	—	—
Public housing	19.8 (16.3–23.7)	—	—	—	—
Multifamily	25.9 (22.0–30.0)	—	—	—	—

Abbreviations: —, does not apply; GED, General Educational Development; HUD, US Department of Housing and Urban Development; IPR, income to poverty ratio; SNAP, Supplemental Nutrition Assistance Program.

a Source: National Center for Health Statistics, National Health and Nutrition Examination Survey, 2005–2018 and linked Housing and Urban Development, 2000–2019.

b Determined by χ^2^ tests.

c Sample sizes are different from sample sizes in Figure 1 because complete case analysis was used and some observations (<5%) were dropped.

d Before 2007, Mexican American people were oversampled. The National Center for Health Statistics recommends not calculating estimates for all Hispanic people for survey periods before 2007 or for Hispanic subgroups other than Mexican American in any survey cycle through 2018 (25).

e “Other” category includes other Hispanic and other race including multiracial.

**Figure 2:**
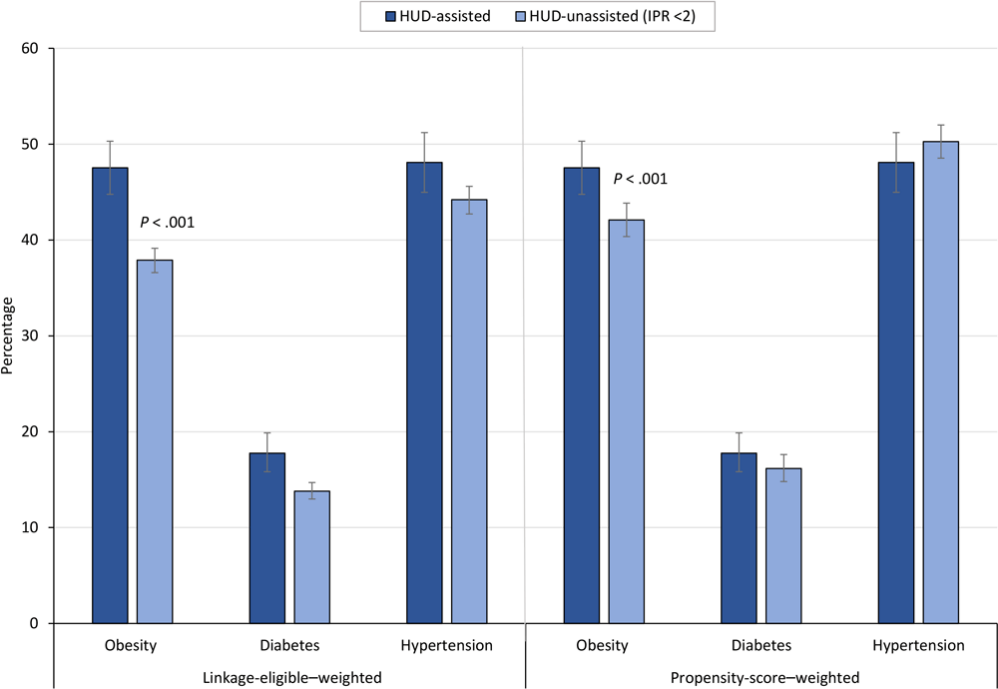
Linkage-eligible–weighted and propensity-score–weighted prevalence of chronic conditions, by housing assistance status, 2005–2018. Error bars indicate 95% CIs. Data source: National Center for Health Statistics, National Health and Nutrition Examination Survey, 2005–2018, and linked data from HUD, 2000–2019. Abbreviations: HUD, US Department of Housing and Urban Development; IPR, income-to-poverty ratio.

The prevalence of obesity among HUD-assisted adults was 47.5% (95% CI, 44.8%–50.3%), diabetes was 17.8% (95% CI, 15.8%–19.9%), and hypertension was 48.1% (95% CI, 45.0%–51.2%) (Figure 2). HUD-unassisted adults with an IPR <2 had a significantly lower propensity-score–weighted prevalence of obesity (42.1%; 95% CI, 40.4%–43.8%) compared with HUD-assisted adults (*P* < .001), but we found no significant differences for diabetes or hypertension.

**Table:** 

Weight	% (95% CI)			
	Condition	HUD-assisted	HUD-unassisted (IPR <2)	*P* value
Linkage-eligible–weighted	Obesity	47.5 (44.8-50.3)	37.9 (36.6-39.1)	<.001
	Diabetes	17.8 (15.8-19.9)	13.8 (13.0-14.7)	.20
	Hypertension	48.1 (45.0-51.2)	44.2 (42.7-45.6)	.26
Propensity-score–weighted	Obesity	47.5 (44.7-50.3)	42.1 (40.4-43.8)	<.001
	Diabetes	17.8 (15.9-19.9)	16.2 (14.8-17.7)	.20
	Hypertension	48.1 (45.0-51.2)	50.3 (48.6-52.0)	.22

HUD-assisted adults had higher odds of obesity (adjusted odds ratio [AOR] = 1.29; 95% CI, 1.12–1.47), but we found no significant differences for diabetes (AOR = 1.16; 95% CI, 0.99–1.36) or hypertension (AOR = 1.00; 95% CI, 0.85–1.18).

In the sensitivity analyses, we found no significant differences in the propensity-score–weighted prevalence of chronic conditions among HUD-assisted adults compared with future HUD-assisted adults for obesity, diabetes, and hypertension (Supplemental Figure 3 [Appendix]). Similar to HUD-unassisted adults with an IPR <2, overall HUD-unassisted adults had a significantly lower propensity-score–weighted prevalence of obesity (41.7%; 95% CI, 40.1%–43.3%), but we found no significant differences for diabetes or hypertension. No significant associations were found between HUD assistance and the 3 chronic conditions in the adjusted propensity-score–weighted logistic regression models that compared HUD-assisted adults and future HUD-assisted adults (Supplemental Figure 4 [Appendix]). When compared with overall HUD-unassisted adults, HUD-assisted adults had higher odds of obesity (AOR = 1.31; 95% CI, 1.14–1.50) and diabetes (AOR = 1.21; 95% CI, 1.03–1.41), but not hypertension. The associations between housing assistance and all 3 chronic conditions were not significant in the analyses of 2013–2018 data with and without disability status (Supplemental Table 3 [Appendix]). Disability status did show positive and significant associations with obesity, diabetes, and hypertension in all models. Including nativity in the model of HUD-unassisted adults with an IPR <2 did not substantially change the results.

## Discussion

In this nationally representative study of adults aged 20 years or older during 2005–2018, almost half of HUD-assisted adults had obesity. HUD-assisted adults had higher adjusted odds of obesity compared with HUD-unassisted adults with an IPR <2. Although populations more likely to receive housing assistance are at higher risk for some chronic conditions, we found no significant associations between HUD assistance and diabetes or hypertension. Two possible reasons for this finding may be the survey years included in our analysis and the large proportion of people with disabilities receiving housing assistance.

Although their study designs were different than ours (in terms of selection into housing assistance, target populations, and comparison group), some studies showed that adults receiving housing assistance had more favorable health outcomes than adults not receiving housing assistance and others showed poorer health and higher prevalence of chronic conditions such as obesity, hypertension, and asthma (8,9,13). A study that used linked NHANES–HUD data from 1999–2016 also showed no differences in diabetes prevalence between people with housing assistance in both public housing and housing choice voucher programs compared with people in the future HUD-assisted group (22). Our study showed no differences in hypertension or diabetes but did show that obesity prevalence was higher among HUD-assisted adults. Findings may have differed across outcomes because the mechanisms explaining associations with housing assistance may vary according to whether the outcomes reflect current health status or longer-term chronic conditions, in relation to acute or longer-term exposures to factors like access to health care, diet and nutrition, stress exposure, physical activity, or others. However, limited information on these longer-term exposures and factors precludes further examination of these pathways.

The 2013–2018 sensitivity analysis showed that associations between housing assistance and obesity were no longer significant with and without controlling for disability status, but the observed associations for 2013–2018 were in the same direction as seen for 2005–2018. A previous study estimated that 44% of the HUD-assisted population were people with disabilities (19). Additionally, a study found that nonelderly adults receiving housing assistance with and without disability insurance and/or supplemental security income were more likely than nonelderly adults not receiving such assistance to have diagnosed chronic conditions (hypertension, asthma, diabetes, and obesity), suggesting health disparities in this population (20). The associations between HUD assistance and obesity may have been larger in earlier periods (before 2013), or smaller sample sizes in the sensitivity analyses using 2013–2018 data could have contributed to nonsignificant estimates, along with disability as a potential confounder. Future analyses with additional years of data after 2018 may be informative in assessing these explanations.

Prior studies on housing assistance and health using national survey data used comparison groups of participants receiving housing assistance 2 years in the future (5,7,22). Our study used a 5-year window from the time of examination to assess recent or future exposure to housing assistance to better account for the nonacute nature of chronic diseases and median duration of housing assistance. Using propensity-score–weighting methods, a HUD-eligible but unassisted comparison was used in this study to adjust for potential confounders and ensure comparability between assisted and unassisted adults to make more robust comparisons. To our knowledge, this is the first study to apply propensity-score–weighting methods to assess federal housing assistance and chronic conditions among adults.

### Limitations

This study has several limitations. The future HUD-assisted group (n = 360) may be considered a better comparison group than groups identified by income because it accounts for various unmeasured factors related to selection into HUD assistance, but the sample was too small to generate precise estimates. Additionally, groups were too small to allow for HUD program-specific analyses or stratified analyses by age, sex, race, or Hispanic origin. Data were also cross-sectional. HUD administrative data were limited to 2019 and earlier, which possibly resulted in missing respondents in the 2015–2016 and 2017–2018 NHANES sample who entered a federal housing program after 2019 and affected the future HUD-assisted group sample size used in the sensitivity analysis. Disability status questions in NHANES were not available before 2013, so they were not included in the 2005–2018 analysis; however, given the high prevalence of disability among the HUD-assisted population, the role of disability status warrants further exploration. Data for duration of housing assistance (or housing stability) were available in the dataset, but these data were not captured in our analyses, which may have attenuated associations because some participants may have had a shorter period of housing assistance than others, limiting the amount of time over which chronic disease prevalence and access to other social services may have been affected. To define hypertension, we used the cutoff of ≥130/80 mm Hg during the entire study period, despite changes in the guideline in 2017. This definition may not have consistently captured data on the use of hypertension medication across all study years, possibly showing an increased use of hypertension medication starting in late 2017. Finally, complete case analysis could have led to bias in the development of the propensity-score weights and final models because respondents with missing covariates were excluded; however, missingness was generally less than 5% overall.

Our study adds to the existing literature that describes associations between federal housing assistance and chronic conditions using nationally representative data on adults aged 20 years or older during 2005–2018. Intervention studies show that changes to neighborhoods and housing environments can reduce high levels of chronic disease, including obesity (13,40). Results from our study can also help inform future research on the relationship between housing assistance and chronic disease. Ongoing linkage of survey and administrative data would ensure sufficient sample sizes for more detailed analyses by subgroups such as housing program and sociodemographic characteristics. The data linkage joins together 2 data sources to answer research questions that could not be answered by either source alone and is likely to be of interest to researchers and policy makers.

## Appendix Supplemental Tables and Figures

The following materials are available in a supplement available upon request from the corresponding author:

Supplemental Figure 1. Distribution of propensity scores for adults aged ≥20 years receiving housing assistance at the time of or in the 5 years before the NHANES examination compared with those who did not receive housing assistance during 2000–2019.

Supplemental Figure 2. Standardized difference in population-weighted means before and after propensity-score weighting: adults aged ≥20 years receiving housing assistance at the time of or in the 5 years before the NHANES examination.

Supplemental Figure 3. Linkage-eligible–weighted and propensity-score–weighted prevalence (95% CIs) of chronic conditions for sensitivity analysis with the future HUD-assisted and overall HUD-unassisted groups, 2005–2018.

Supplemental Figure 4. Adjusted propensity-score–weighted associations between housing assistance status and chronic conditions, sensitivity analysis, 2005–2018.

Supplemental Figure 5. Flowchart of analytic subsample for sensitivity analysis, 2013–2018.

Supplemental Table 1. Linkage-eligible–weighted and propensity-score–weighted selected demographic and household characteristics of US adults (aged ≥20 years), by housing assistance status at the time of NHANES examination for sensitivity analysis with the future HUD-assisted and overall HUD-unassisted groups, 2005–2018.

Supplemental Table 2. Linkage-eligible–weighted and propensity-score–weighted selected demographic and household characteristics of US adults (aged ≥20 years), by housing assistance status at the time of NHANES interview, 2013–2018.

Supplemental Table 3. Adjusted propensity-score–weighted associations between housing assistance status, disability status, and chronic conditions, 2013–2018.
